# Understanding mechanistic responses underlying diurnal photoprotection and photosynthetic plasticity among cacao genotypes under natural amazonian field conditions

**DOI:** 10.1371/journal.pone.0351655

**Published:** 2026-06-11

**Authors:** Juan Carlos Suárez, José Iván Vanegas, Fabricio Eulalio Leite Carvalho

**Affiliations:** 1 Programa de Ingeniería Agroecológica, Facultad de Ingeniería, Universidad de la Amazonia, Florencia, Colombia; 2 Programa de Maestría en Sistemas Sostenibles de Producción, Facultad de Ciencias Agropecuarias, Universidad de la Amazonia, Florencia, Colombia; 3 Programa de Doctorado en Ciencias Naturales y Desarrollo Sustentable, Facultad de Ciencias Agropecuarias, Universidad de la Amazonia, Florencia, Colombia; 4 Grupo de Investigaciones Agroecosistemas y Conservación en Bosques Amazónicos-GAIA, Centro de Investigaciones Amazónicas CIMAZ Macagual César Augusto Estrada González, Florencia, Colombia; 5 Corporación Colombiana de Investigación Agropecuaria (Agrosavia), Centro de Investigación La Suiza, Rionegro, Colombia; University of Innsbruck, AUSTRIA

## Abstract

Cacao (*Theobroma cacao*) is a key tree species for chocolate production and a vital income source for millions, especially in Africa and South America. In the Amazon, physiological studies on cocoa have focused on abiotic factors, but the comparison of clones in these environments is still in its infancy.This study examined the physiological responses of cacao clones to the typical diurnal conditions of the Colombian Amazon. We measured environmental, plant health, and chlorophyll fluorescence variables in 18 cacao clones using MultispeQ and Imaging-PAM throughout the day; we estimated F_v_/F_m_, ΦII, ETR, and NPQ_t_, calculated SLA, and quantified photosynthetic pigments. Significant phenotypic differences were identified in photosynthetic efficiency and morphology. High specific leaf area (SLA), linked to efficient energy capture and reduced transpiration, was observed in LUKER-40 and FGH-4. Chlorophyll *a* fluorescence approach, revealed differences in photochemical efficiency (F_v_/F_m_) and excess energy dissipation. Genotypes like FGH-4, FSV-41, and IMC-67 showed high F_v_/F_m_ and electron transport rates (ETR) (p < 0.05) under heat stress, indicating strong PSII functionality. Conversely, ICS-95 relied on non-photochemical quenching (NPQ_t_) for heat dissipation. LUKER-50, with high carotenoid content, demonstrated resilience to oxidative stress. Variations in chlorophyll content affected light capture efficiency, with ICS-60 excelling in photosynthesis under high temperatures and excess light at noon. Leaf temperature regulation emerged as critical, with genotypes like LUKER-40 maintaining cooler leaves to avoid potential thermal damage. These traits highlight the acclimation of genotypes to high light and heat in the Colombian Amazon, with LUKER-50, ICS-95, FLE-3, and LUKER-40 identified as promising candidates for agroforestry in this region.

## 1 Introduction

Cacao (*Theobroma cacao* L.) is a tropical perennial species native to the Amazon basin [1] and stands out as one of the most important cash crops worldwide, representing the economic livelihood for small farmers in tropical regions in West Africa, Southeast Asia, and Latin America [2,3]. The seeds (cocoa beans) are the primary raw material for chocolate production and for the chocolate, cosmetics, and pharmaceutical industries [4]. Its production is estimated at 5.175 million tons of beans for 2020–2021 from Africa 77%, America 17%, and only the remaining 5% is occupied by Asia and Oceania [5]. Similarly, an increase in production is expected to be 7.3% by 2025 [6]. Cacao exhibits excellent genetic variability, with ten genetic groups found (Marañón, Curaray, Criollo, Iquitos, Nanay, Contamana, Amelonado, Purús, Nacional y Guianna) in the Amazon [7,8].

Despite its importance, cacao cultivation in different regions of the world faces great challenges in relation to the effect of climate variability on the yield and sustainability of the crop [9–13] as variations in both rainfall and temperature are expected which may impact crop phenology [14]. In this sense, average global temperatures are expected to increase between 1.4 and 4.8 °C in the next years [14], a condition that will affect the design of agroforestry systems [15]. In order to achieve optimal microclimatic conditions for cocoa cultivation, these agroforestry systems are likely to increase shade density, which may reduce light availability and, consequently, impact photosynthetic activity [16,17].

Different studies [9,17–19] have researched the functioning of the photosynthetic apparatus in cacao and have identified genotypes with physiological and agronomic traits to face different light and temperature conditions. Indeed, a key objective of breeding programs is to identify genotypes that are resistant to environmental fluctuations by monitoring their physiological responses under adverse conditions [20]. Traits related to physiological behavior allow the identification of the effects of environmental conditions on the photosynthetic apparatus [21]. In this sense, chlorophyll *a* (Chl_a_) fluorescence represents a unique tool for diagnosing plant health status, photosynthetic performance, and effects of biotic or abiotic stress in plants [22]. Knowledge of this response would help maximize yield and minimize time losses, understanding the potential of cacao clone’s acclimation to different environments.

When assessing the acclimatization potential of different genetic materials to a given environment, it is essential to recognize the distinction between natural environments—characterized by high variability—and laboratory conditions, which are more stable and thus conform to simplified models. On a typical day, several factors can affect plants differently, such as temperature, intensity and duration of light, relative humidity and vapor pressure deficit, in addition to variations in the mechanisms underlying the circadian cycle response between different species and varieties. In fact, adverse abiotic conditions are the rule rather than the exception under field conditions [23]. During a typical day, changes in environmental stimuli, especially light, temperature and humidity, are closely related to changes in gene expression and accumulation of key metabolites for the regulation of photosynthesis. For example, the xanthophyll cycle, widely studied in different plant species, presents strong regulation as a function of the time of day [24]. It is important to highlight that the integration of these stimuli with the intrinsic response capacity of each species or variety is fundamental to determine the observed phenotypic response. Therefore, for more effective results in a breeding program, comparing the performance of different varieties under fluctuating environmental conditions of a typical day is a recommended approach.

Working with coffee plants, Toniutti et al. [25] showed an alteration of the amplitude of circadian clock genes in a hybrid material and reported a correlation with increased photosynthetic electron transport efficiency, which could explain better agronomic performance when compared with a standard variety. In cocoa, González-Ceballos et al. [26] evaluated gas exchange parameters in five genotypes grown under agroforestry system every hour from 08:00h to 17:00 h., indicating that except for CCN-51, all cocoa genotypes reached peak photosynthesis between 08:00 h and 09:00 h. At noon, rates declined across clones, notably and in the afternoon, the genotype TCS-19 remained stable, while TCS-13 and CCN-51 showed a recovery. Nevertheless, only few studies have investigated deeper the physiological responses of different cocoa varieties in a typical day analysis, especially in the amazon region.

In the Amazon, unlike other cacao-producing areas in Colombia (Santander, Arauca, and Huila), the annual average radiation is only 3–4 hours of sunlight per day [27] due to its high cloud cover. However, frequently the maximum photosynthetic photon flux density can overcome 2,000 μmol m^-2^ s^-1^ at noon. This specific typical day situation has caused variations in the microclimatic conditions [28] and modification of the suggested agroforestry arrangement [29]. Moreover, the dayly in the Amazon region can reach up to 35°C, a situation that can have a negative impact on the functioning of the photosynthetic apparatus [30]. Since the diurnal temperature is well above the optimal range (26 and 32 ºC) for cacao growth, it is necessary to determine the effects of dayly in the Amazon on the physiological behavior of the cacao [31–33].

Photoprotective mechanisms—particularly non-photochemical quenching (NPQ), which dissipates excess light energy as heat, and photosystem I (PSI) oxidation, which prevents over-reduction of the electron transport chain—are critical for tolerance to light and heat stress in plants. Given the extreme diurnal fluctuations of light and temperature in the Amazon, it is essential to evaluate how these processes vary among cacao genotypes over the course of a day. Therefore, the aim of this study is to compare the diurnal response of the photosynthetic apparatus (including Chl_a_ fluorescence, NPQ, and PSI oxidation) in 18 cacao cultivars, to identify physiological traits associated with greater photoprotection and photosynthetic recovery under high irradiation and thermal stress. These findings will inform clone selection for breeding programs and the design of agroforestry systems adapted to Amazonian conditions.

## 2 Materials and methods

### 2.1 Plant material and experimental design

The experiment was carried out during the period of minimum rainfall from November 2020 to February 2021 at the Amazon Research Center CIMAZ Macagual of the University of Amazonia (1°37'N and 75°36'W at 360 m.a.s.l.), located in Florencia, Caquetá (Colombia). The center is in a tropical rainforest ecosystem with a mean annual precipitation of 3,800 mm and a mean annual temperature of 26.48 °C with an increase of 2.35 °C temperature with reference to the mean daily temperature of 25.82 °C for the year 1981—the relative humidity of 84% and 1,200 hours of sunshine per year^-1^ [34]. Measurements were made on 18 cacao clones, eight of which were national clones resulting from the genetic improvement program of the National Federation of Cacao Growers of Colombia (FEDECACAO) (FEAR-5, FEC-2, FGH-4, FLE-3, FSA-12, FSA-13, FSV-155 and FSV-41) and two clones from LUKER (LUKER-40 and LUKER-50), five clones from Trinidad being four from Imperial College Selection (ICS-1, ICS-39, ICS-60, ICS-95) Trinidadian hybrid and one from Trinidad Selection Hybrid (TSH-565), two clones belong to Estación Experimental Tropical de Costa Rica (EET-8), and one clone from the Castro Naranjal Collection of Ecuador (CCN-51) and finally the Amazonian Iquitos Mixed Calabacillo (IMC-67) from Peru. The clones were chosen for their wide distribution, use in the Amazon region, regional representativeness, and productive relevance. Each of the cacao clones was grafted on plants of clone IMC-67 for three months of growth in the nursery. After six months of graft development, each seedling was transplanted into a 100 L plastic bag with a soil mixture of clay, sand, and organic substrate in a 3:1:1 ratio. Plants of each clone (n = 30) were acclimatized for 180 days under a radiation level of 600 µmol m^-2^ s^-1^ condition generated using a poly-shade net (Colmallas®), had a constant water availability close to field capacity (ΨL ~ −0.2 ± 0.01 MPa MPS-2 Decagon Devices, Inc). A completely randomized plot design with three replications was used for the study. The plot was composed of ten plants for each cacao clone.

### 2.2 Measurements of photosynthetic energy use and leaf cooling under high-temperature stress

Measurements related to environmental variables and photosynthetic characteristics based on chlorophyll fluorescence were collected using the portable device (MultispeQ v2, PhotosynQ Inc., East Lansing, MI, USA) connected to the PhotosynQ app via Bluetooth using a Poco X3Pro Android smartphone (Xiaomi Technology Co., Ltd., Beijing, China). The default Photosynthesis RIDES non-open/close protocol developed on the PhotosynQ platform [35] was used for measurements. The device features a PAR sensor on the top (TCS34715FN, AMS-TAOS, USA), humidity, and temperature sensors (BME280, BOSCH, USA) to the right of the clamp, and a small infrared thermometer located at the bottom to measure the temperature of the leaf (Temperature Sensor Digital, Infrared MLX90615SSG-DAG-000-TU, Malaysia). The device also has photodiodes above and below the clamp to measure absorbance at 450, 535, 605, 650, 730, 850, and 940 nanometers. The cacao plants were monitored at phenological stage BBCH 19 [36], corresponding to the presence of nine fully expanded true leaves on the main shoot [36]. Measurements were taken on fully expanded mature leaves, free from insect damage and located between the second and fourth leaf from the apex of each plant, as previously described by Suárez et al. [16,17], 180 days after grafting. The leaves were monitored at seven different times (06:00 h, 08:00 h, 10:00 h, 12:00 h, 14:00 h, 16:00 h, and 18:00 h), with three leaves per plant and four plants per clone. Measurements were made between 06:00 h and 08:00 h to assess and track the possibility of early stress in the plants, which is when evaporative demand is low, at 12:00 h and 14:00 h to assess the possible effect of maximum daily thermal stress from radiation and temperature, and at 18:00 h to track the daily recovery power of the cocoa clones, which is when irradiance and temperature decrease. Measurements were taken in the middle of the morning at 10:00 h and in the early afternoon at 16:00 h to characterize the transition between these periods. This process was carried out for three consecutive days. Before starting the monitoring, both the plants and the leaves were marked and labeled with fluorescent embroidery thread and masking tape at the base of the stem of the plants and the base of the petiole of the leaves.

The photosynthesis RIDES non-open/close protocol allowed monitoring of (i) abiotic factors including temperature and relative humidity, (iii) leaf morphological attributes, (iv) chlorophyll fluorescence parameters, and (v) parameters based on absorbance at various wavelengths. The variables measured were ambient temperature, leaf temperature (LT), and leaf temperature differential (LTD), calculated using the difference between leaf temperature and ambient temperature using the various sensors located on the MultispeQ v2. Various chlorophyll fluorescence parameters were calculated based on previous data (F_o_: Minimal fluorescence yield of dark-adapted sample, F_m_: Maximal fluorescence yield of dark-adapted sample, F_s_: Steady-state fluorescence, F_o_’: Minimal fluorescence yield of dark-adapted sample, F_m_’: Maximal fluorescence yield of illuminated sample) such as the photochemical quantum yield of photosystem II (ΦII) where $\Phi II=\;\frac{Fm^{\prime }-\;Fs}{Fm^{\prime }}$ [37], the unregulated energy quantum yield of photosystem II (ΦNO) where $\Phi NO=\;\frac{Fs}{Fm}$ and the non-photochemical quenching quantum yield of photosystem II (ΦNPQ) where ΦNPQ=1−ΦII− ΦNO [35]; these three, when added together, give 1. Likewise, the maximum quantum efficiency of photosystem II (F_v_/F_m_) understood as $F_{v}/F_{m}=\;\frac{Fm-\;Fo}{Fm}$ [37]. The total non-photochemical quenching (NPQt) where $NPQ=\;\frac{Fm\;-\;Fm^{\prime }}{Fm^{\prime }}$ [37] and the fraction of photosystem II (qL) where $qL=qP\times \;\frac{F{\text{0}}^{\prime }}{Fs}$ [38] open centers were also determined. Linear electron transport (LEF) understood as LEF= ΦII×PAR×0.4, which indicates the amount of energy that moves through the chloroplast and is mainly related to photosynthetic activity, was measured [35].

Among the absorbance-based parameters, the amplitude of the electrochromic band displacement signal was determined (ESC_tau) [36] using the dark interval relaxation kinetics (DIRK) of the electrochromic displacement [39]. The ESCt estimates transtilacoid membrane proton transport in relation to proton motive force (*pmf*) and ECSt_mAU the ATP synthase capacity. In addition, the proton conductivity of ATP synthetase (gH+) and the steady-state proton flux (vH+) were calculated. Different redox states of PSI were also estimated from those described by Kanazawa et al. [36], corresponding to the total number of active PSI sites (PSI_act_), the fraction of oxidized PSI sites (PSI_ox_) and in the open state (PSI_open_), as well as the reduced PSI fraction (PSI_or_) corresponding to the lateral restrictions of the PSI acceptor resulting from the accumulation of electrons in the PSI acceptors during steady-state illumination. Similarly, the electron transfer rate constant in P700 (_k_P700), the initial steady-state P700 electron transfer rate (_i_P700), and the oxidized P700 electron transfer lifetime (tp700) and probability of P700 oxidation after one-photon absorption (P700_DIRK_) were estimated.

### 2.3 Specific leaf area and photosynthetic pigments

Specific leaf area (SLA) was determined using seven leaf discs of (3.14 cm^2^), omitting the middle vein of each leaf previously used to measure Chl_a_ fluorescence (n = 1152 corresponding to 4 plants per genotype (18 clones), four leaves per plant and 16 discs per plant). The discs were dried at 70 °C until a constant mass was reached, and the SLA was determined as the ratio of leaf disc area to their respective dry mass [40]. The protocol described by Lichtenthaler [41] was followed to calculate pigments in cacao clones. In this regard, the leaves of the plants previously used to calculate SLA had seven leaf discs (3.14 cm²) extracted to estimate the levels of chlorophyll a (Chl_a_), chlorophyll b (Chl_b_), total chlorophyll (Chl_t_), and carotenoids (Car). For ${Chl}_{a}=\text{1}\text{2}.\text{2}\text{5}\;\times (\text{A663})-\text{2}.\text{79}\times (\text{A647})$, ${Chl}_{b}=\text{ 21}.\text{5}\times (\text{A647})-\text{5}.\text{1}\times (\text{A663})$, ${Chl}_{t}=\;\text{7}.\text{15}\times (\text{A663})+\text{18}.\text{71}\times (\text{A647})$ y $Car=\;\frac{\left(\text{1000}\times \text{A470})-\text{1}.\text{82}\times {Chl}_{a})-(\text{85},\text{02}\times {Chl}_{b}\right)}{\text{1}\text{9}\text{8}}\;$, where A denotes the absorbance at the specified wavelength (nm). Subsequently, the calculated concentrations were multiplied by the final extract volume (in liters) and divided by the initial mass of plant material used and were expressed as g kg^−1^ dry matter (DM).

### 2.4 Data analysis

To analyze each of the variables, a linear mixed model (LMM) was fitted. The factors of cocoa clone and time, as well as the interaction between these factors, were declared as fixed effects. Day, plant, and leaf were considered random effects. The assumptions of the LMM (normality and homoscedasticity) were evaluated with a Q-Q plot and a plot of residuals against predicted values, respectively. The variables were analyzed using a linear mixed model (LMM) which follows the following model: $Y_{ijklm}=\mu +a_{i}+\beta_{j}+(\alpha \beta {)}_{ij}+d_{k}+p_{l(k)}+h_{m(kl)}+\varepsilon_{ijklm}$, Where: $Y_{ijklm}$: Each of the physiological variables, μ: Overall mean, $\alpha_{i}$ = Fixed effect of the i-th cocoa clone, $\beta_{j}$ = Fixed effect of the j-th hour, $(\alpha \beta {)}_{ij}$ = Fixed interaction clone × hour, $d_{k}$ = Effect of the k-th day, $p_{lk}$ = Effect of the l-th nested plant per day, $h_{mkl}\;$= Random effect of the m-th nested leaf in plant and day, $\varepsilon_{ijklm}$ = Experimental error. The Di Rienzo, Guzmán and Casanoves (DGC) test [40] was used to determine the difference in means of the fixed factors using a 5% error. Box plots were used to represent SLA and leaf thickness. The F_0_, F_m_, and F_v_/F_m_ values were represented by a bar diagram and the others by a line diagram. A circular cluster analysis was performed using the data regarding photosynthetic traits to identify groups of cacao clones with remarkable performance. Similarly, principal component analysis (PCA) was used to identify the relationship of cacao clones with photosynthetic traits, and a Monte-Carlo permutation test accompanied this. A Pearson correlation analysis and “*corrplot*” packages were performed to determine the association between environmental variables and photosynthetic traits of chlorophyll fluorescence. The LMMs were performed with the *lme* function of the nlme package, clusters with the “*cluster*” and “*NbClust*” packages, and graphical outputs were performed in the “*ade4*,” “ggplot2,” “factoextra,” and “dplyr” packages of the R language software, version 4.5.1 [42].

## 3 Results

**Fig 1:**
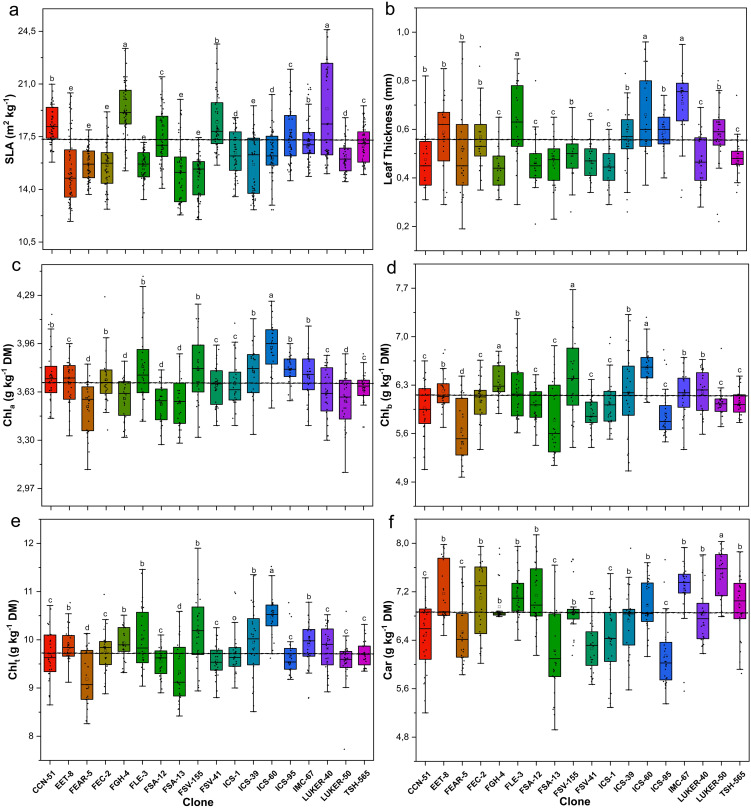
Box plots of the scatter plot for physiological leaf characteristics in terms of a. specific leaf area (SLA); b. leaf thickness; c. chlorophyll a (Chl_a_); d. chlorophyll b (Chl_b_); e. total chlorophyll (Chl_t_) and f. carotenoid (Car) of different clones of *Theobroma cacao* L. In the boxplots, different letters above the boxes indicate statistically significant differences between group means based on the DGC test at a 5% significance level. The boxes represent the interquartile range (IQR), the horizontal line inside each box indicates the median, and the whiskers show the minimum and maximum values excluding outliers.

Overall, no statistical difference was found between clone interaction and monitoring time for the variables leaf thickness, carotenoids, PSI active centers (PSI_act_), the PSI oxidized (PSI_ox_), rate constant of P700 electron transfer (_k_P700) and lifetime of P700 electron transfer (_t_P700) (S4 File). Leaf-level traits (SLA and thickness) showed differences at the clone level (P < 0.05, Fig 1a). In general, the average SLA among clones was 16.80 ± 0.25 m^2^ kg^-1^, where LUKER-40 (19.35 ± 0.43 m^2^ kg^-1^) and FGH-4 (19.1 ± 0.24 m^2^ kg^-1^) had the highest values (P < 0.05, Fig 1a). Contrary to the EET-8 (15.3 ± 0.11 m^2^ kg^-1^), FEAR-5 (15.7 ± 0.11 m^2^ kg^-1^), FEC-2 (15.6 ± 0.14 m^2^ kg^-1^), FLE-3 (15.6 ± 0.12 m^2^ kg^-1^), FSA-13 (15.1 ± 0.27 m^2^ kg^-1^) and FSV-155 (14.9 ± 0.18 m^2^ kg^-1^) clones, which presented the lowest averages. The mean cacao leaf thickness was 0.56 ± 0.03 mm, where the clones with the greatest thickness corresponded to FLE-3 (0.69 ± 0.04 mm), IMC-67 (0.73 ± 0.03 mm), and ICS-60 (0.68 ± 0.03 mm) (P < 0.05, Fig 1b). The Chl_a_ content varied between 3.08 and 4.42 μg cm^-2^ with an average of 3.7 ± 0.01 μg cm^-2^ where the clone with the highest content was ICS-60 with an average of 3.93 ± 0.03 μg cm^-2^, however clones such as FEAR-5 (3.56 ± 0.03 μg cm^-2^), FGH-4 (3.60 ± 0.03 μg cm^-2^), FSV-12 (3.54 ± 0.03 μg cm^-2^), FSV-13 (3.56 ± 0.03 μg cm^-2^) and LUKER-50 (3.55 ± 0.03 μg cm^-2^) presented the lowest values. As for Chl_b_ content, it ranged from 4.46 to 7.78 μg cm^-2^ with an average among clones of 6.09 ± 0.02 μg cm^-2^ where clones FGH-4 (6.38 ± 0.07 μg cm^-2^), FSV-155 (6.38 ± 0.07 μg cm^-2^) and ICS-60 (6.52 ± 0.07 μg cm^-2^) presented the highest values, while FEAR-5 (5.67 ± 0.06 μg cm^-2^) had the lowest value. The mean carotenoid content in the leaves of the cacao clones was 6.79 ± 0.03 μg cm^-2^ and ranged from 4.63 to 8.14 μg cm^-2^. The clone with the highest carotenoid content was LUKER-50. However, different clones, including FSA-13, ICS-95, and FSV-41, had the lowest carotenoid content values. Leaf chlorophyll content ranged from 27.3 to 66.7 SPAD, averaging 46.6 ± 0.3 SPAD. Cacao clones FGH-4 (55.57 ± 0.75 SPAD) and FSV-155 (52.83 ± 1.40 SPAD) showed the highest values compared to the other clones. However, clone ICS-95 presented the lowest SPAD chlorophyll value (37.77 ± 0.84 SPAD).

**Fig 2:**
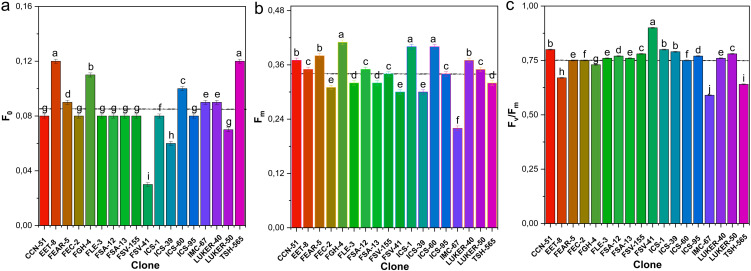
Chlorophyll fluorescence parameters taken in fully developed leaves at predawn: **a.** Initial fluorescence (F_0_), **b.** Maximum fluorescence **(F**_**m**_), **c.** Maximal PSII quantum yield (F_v_/F_m_) in different clones of *Theobroma cacao* **L.** The mean and error bars correspond to 32 AOI (area of interest) for each clone, i.e., 8 AOI were taken for each leaf, four plants per clone, one leaf per plant. The selected leaf started from the beginning fully expanded, healthy with no visible physical damage. The change in the color gradient from violet to red indicates the change in value from higher to lower for each variable.

Chlorophyll fluorescence parameters showed significant differences between clones (P < 0.001, Fig 2) in initial fluorescence (F_0_), maximum fluorescence (F_m_), and maximum photochemical quantum yield of PSII (F_v_/F_m_). The F_v_/F_m_ ranged between 0.59 and 0.90, where clone FSV-41 presented a value higher than 0.82; the opposite was the case for most of the clones, which presented stress induced by the increase in temperature (Fig 2). Clones FGH-4, ICS-60, and ICS-1 presented a greater magnitude in F_m_ values; however, the magnitude in F_0_ values was similar in cacao clones, with higher values being more evident in clones EET-8, TSH-565, and FGH-4 (Fig 2). When analyzing the Chl_a_ in the color gradient for the fluorescence parameters in the dark adaptation phase, it was found that clones with a tendency from orange to red in the leaves represent stressed plants, specifically in F_0_ (S1 Fig) in clones FSV-41 and ICS-39. In F_m_, it was found that clone IMC-67 showed a yellow color, and clones such as FGH-4 and ICS-60 lines evidenced higher values of 0.41 and 0.40, respectively, represented in the green, fluorescent color. The F_v_/F_m_ was significantly reduced in clones such as IMC-67, TSH-565, and EET-8, a condition that can be observed in the gradient changing from violet and blue to light green (S1 Fig).

**Fig 3:**
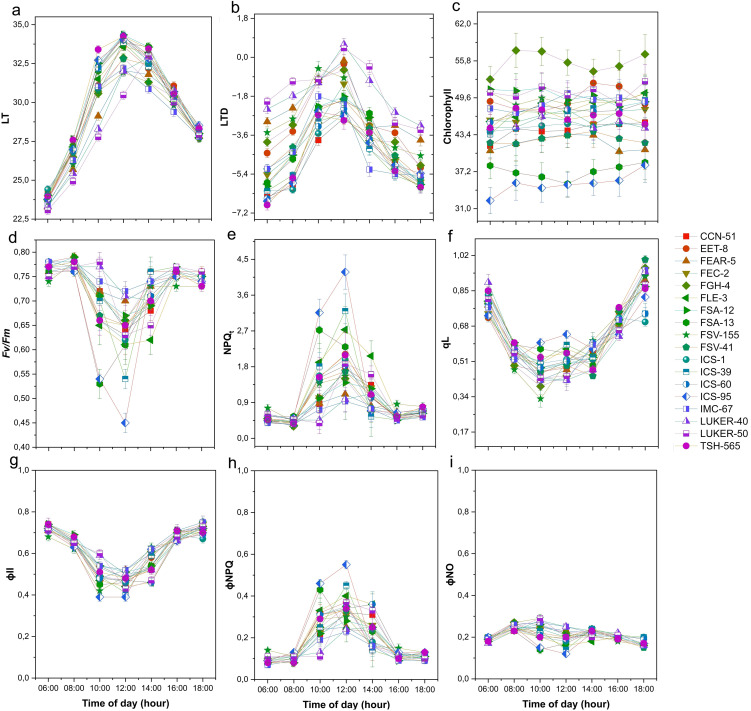
Diurnal variation of environmental and photosynthetic traits in different clones of *Theobroma cacao* L. **a.** Leaf Temperature (LT); **b.** Leaf Temperature Difference (LTD); **c.** Chlorophyll; **d.** Maximal PSII quantum yield (F_v_/F_m_); **e.** Total non-photochemical quenching (NPQ_t_); **f.** Fraction of PSII open centers (qL); **g.** quantum yield of PSII (ΦII); **h.** Fraction of light dedicated to non-photochemical quenching (ΦNPQ); **i.** Fraction of light lost via nonregulated photosynthesis inhibitor processes (ΦNO) in cocoa clones.

Cacao clones at different times of the day showed significant statistical differences (P < 0.05) for all traits such as LT, LTD, SPAD, F_v_/F_m_, NPQt, qL, ΦII, ΦNPQ, and ΦNO. The LT and LTD showed an increase between 6 and 12 hours, followed by a progressive decrease towards sunset. During the monitoring period, the ambient temperature ranged from 28.8 ± 0.2 °C at 06:00 h to 35.5 ± 0.1 °C at 14:00 h, a condition that significantly affected the leaf temperature of the clones evaluated in the different samples taken during the day (P < 0.05). For example, LUKER-50 and LUKER-40 presented the lowest leaf temperature compared to the other clones during the different samplings made during the day (Fig 3a). The opposite situation occurred for clones TSH-565, CCN-51, and ICS-1, which had the highest leaf temperatures (Fig 3a). In the case of LTD, it ranged from 1.6 to −7.2 °C during the monitoring period. It showed differences at the clone level in the setting of the hourly temperature in reference to the ambient temperature. For example, at 06:00 h (28.8 ± 0.2 °C), LTD was −2.05 °C for LUKER-50 to −6.83 °C for TSH-565, reaching a value above the ambient temperature (0.6 °C) for LUKER-50, (Fig 3b) at 14:00 h by the time the other clones set leaf temperature below ambient temperature.

**Fig 4:**
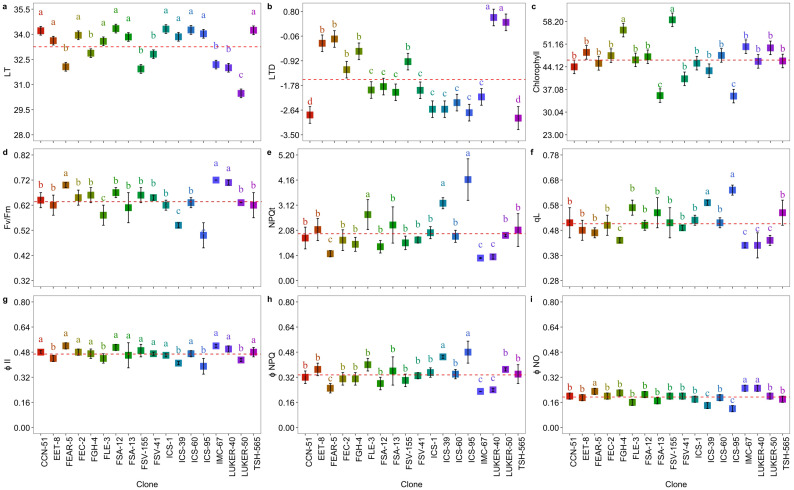
Environmental and photosynthetic traits in different clones of *Theobroma cacao* L. at 12:00 h of the day. **a.** Leaf temperature (LT); **b.** Leaf temperature difference (LTD); **c.** Chlorophyll; **d.** Maximum PSII quantum yield (F_v_/F_m_); **e.** Total non-photochemical quenching (NPQ_t_); **f.** Fraction of PSII open centers (qL); **g.** Quantum yield of PSII (ΦII); **h.** Fraction of light devoted to non-photochemical quenching (ΦNPQ); **i.** Fraction of light lost through unregulated photosynthesis inhibitory processes (ΦNO) in cocoa clones.

The SPAD chlorophyll index varied depending on the time of day. Values higher than the average (46.13 ± 2.46) were evident in clone FGH-4 (55.27 ± 0.69) and lower values in clones ICS-95 and FSA-13 (35.21 ± 0.67 and 37.05 ± 0.46) respectively (Fig 3c). Regarding the quantum efficiency of the PSII, normal behavior was found without stress conditions between 06:00 h and 08:00 h, a period in which F_v_/F_m_ oscillated between 0.74 and 0.78 (Fig 3d). However, at 10:00 h, there was an increase of 4.5 °C in the environmental temperature with reference to that presented at 06:00 h, where the FSA-13 and ICS-95 clones presented F_v_/F_m_ values below 0.54 (Fig 3d). The increase in temperature proportionally affected NPQ_t_, a situation that was evident in clones ICS-39 and ICS-95 whose NPQ_t_ values rose to 3.20 ± 0.40 and 4.28 ± 0.58, respectively (Fig 3e). The fraction of PSII open centers (qL) showed a behavior inversely proportional to the increase in temperature, that is, when increasing the temperature by 4.5 °C, the qL value went from 0.79 at 06:00 h to 0.46 at 10:00 h. At the clone level, ICS-95 showed an increase at noon (P < 0.05, Fig 3f). At the energy uptake pathway level, it was found that the energy uptake was higher in all clones in ΦII compared to the other pathways (ΦNPQ, ΦNO, Fig 3g, 3h, 3i). The ΦII fraction decreased with increasing temperature during the day (Fig 3g), and this excess energy was dissipated as heat (ΦNPQ, Fig 3h), a process in which the ICS-95 and ICS-39 clones increased heat dissipation. The unregulated fraction (ΦNO) varied significantly in the middle of the day when the ambient temperature was higher (Fig 3i). Likewise, higher maximum quantum efficiency of photosystem II F_v_/F_m_, ΦII and ΦNO at 12:00 h was evident in FEAR-5, LUKER-40 and IMC-67 clones (Fig 4d, 4g, 4i). The increase is these pathways caused a lower energy distribution for the ΦNPQ and NPQ_t_ thermal pathways (Fig 4h, 4e).

**Fig 5:**
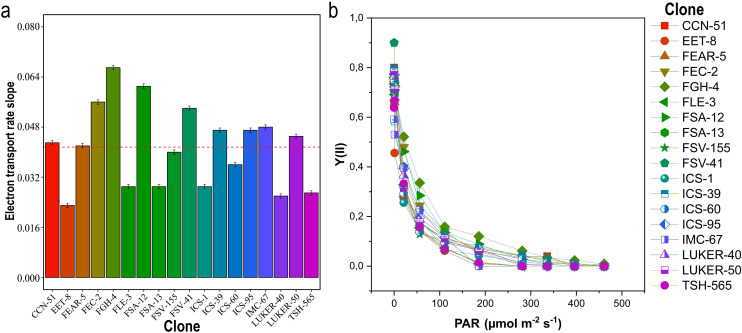
Photosynthetic responses as a function of photosynthetically active radiation (PAR) levels of different clones of *Theobroma cacao* L. Fast light curves as a function of PAR: **(a)** Effective quantum yield of PSII (Y(II)); **(b)** Electron transfer rate slope. Mean and error bars correspond to 32 AOI (area of interest) for each clone, i.e., 8 AOI were taken for each leaf, four plants per clone, one leaf per plant. The selected leaf started from the beginning fully expanded, healthy with no visible physical damage.

We found that Y(II) decreased with increasing levels of radiation (PAR) in each of the different cacao clones evaluated (Fig 5). Clones FGH-4, CCN-51, and ICS-1 showed a higher level of Y(II) (S2 Fig), showing a higher color gradient from 0.08 to 460 μmol m^-2^ s^-1^ of PAR for FGH-4, similarly in the quantitative analysis of Y(II) significant differences between clones were evident (P < 0.05, Fig 5a). The slope of the curve relating electron transfer rate (ETR) to photosynthetically active radiation (PAR) varied among the clones evaluated. Fifty-five. 55% of the clones showed a slope greater than 0.041, with clones FGH-4, FSA-12, FEC-2, FSV-41, IMC-67, ICS-39, ICS-95, LUKER-50, CCN-51 and FEAR-5 standing out among them. In contrast, the remaining 44.44% showed slopes below this value, including clones such as FSV-155, ICS-60, FLE-3, FSA-13, ICS-1, TSH-565, LUKER-40 and EET-8, suggesting differences in electron transfer rate efficiency as radiation increases (Fig 5b).

**Fig 6:**
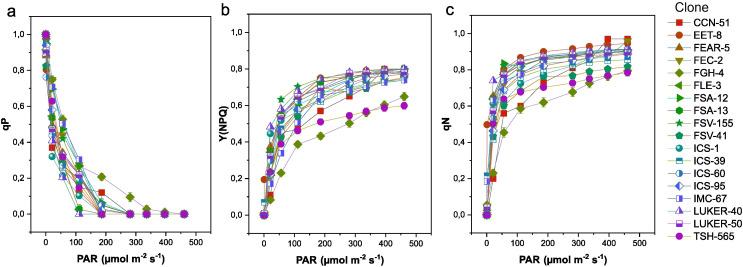
Photosynthetic responses as a function of photosynthetically active radiation (PAR) levels of different clones of *Theobroma cacao* L. Fast light curves as a function of PAR: **a.** Coefficient of photochemical quenching (qP); **b.** Quantum yield of regulated energy dissipation (Y(NPQ)); **c.** Coefficient of non-photochemical quenching (qN). Mean and error bars correspond to 32 AOI (area of interest) for each clone, i.e., 8 AOI were taken for each leaf, four plants per clone, one leaf per plant. The selected leaf started from the beginning fully expanded, healthy with no visible physical damage.

For the photochemical extinction coefficient (qP) level, a decrease in the blue, light green, and black gradient was found. This decrease in qP was related to the increase in PAR (S3 Fig). Among the cacao clones, FGH-4 had the highest fraction of energy devoted to photochemical extinction (qP, P < 0.05, Fig 6a). Similarly, CCN-51 showed high qP values, evidencing a lower loss in the photosynthetic apparatus during the fast light curve (S3 Fig). Therefore, those cacao clones that reduced their qP energy fraction increased the performance in dissipating energy as heat (Y(NPQ), Fig 6b) or unregulated processes (qN, Fig 6c). In these cases, the color gradients for (qN, S3 Fig) in cacao leaves with reductions in qP tended toward violet while in leaves with increases in qP, the qN value increased as the color gradient changed from green to blue from the 20 μmol m^-2^ s^-1^ PAR level, similar condition was observed for Y(NPQ). Thus, cacao clones such as FGH-4 and TSH-565 presented the lowest values (Y(NPQ), P < 0.05, Fig 6b).

**Fig 7:**
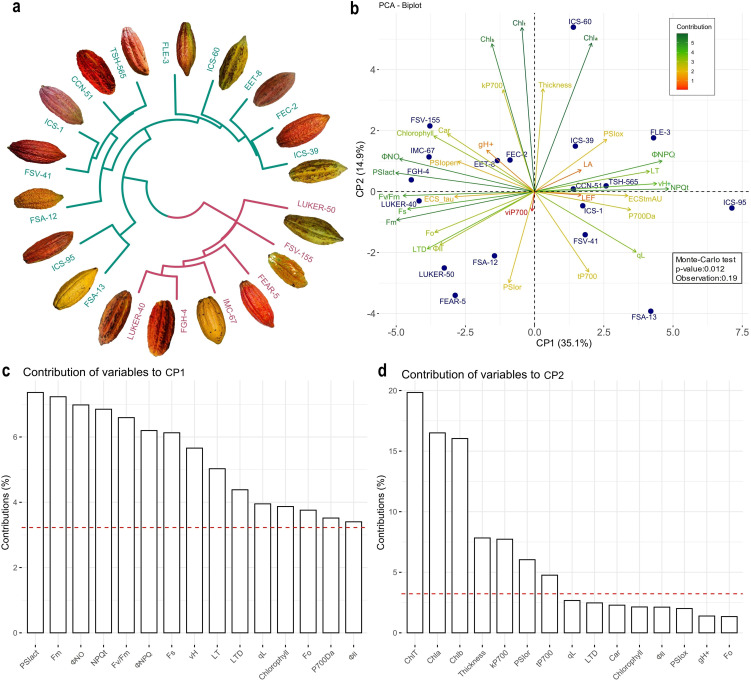
Cluster and principal component analysis of energy use and distribution in the photosynthetic apparatus in different cocoa clones. **(a)**. Cluster analysis (Ward's method, Euclidean distance). **(b)**. Correlation circle between variables of photosynthetic energy use and distribution, from green to red color from higher to lower contribution. (c, d) contribution of physiological variables to the formation of the CP1/CP2 principal components of the PCA; variables above the red line in figure c and d contribute significantly to each principal component. The color of the vectors in the gradients from green to red indicates their contribution (%) from highest to lowest to the component. LT: leaf temperature; LTD: leaf differential temperature; LA: leaf angle; Thickness: leaf thickness; Chlorophyll: relative chlorophyll; Chl_a_: chlorophyll a; Chl_b_: chlorophyll b; Chl_t_: total chlorophyll; Car: carotenoids; ΦII: photochemical quantum yield of PSII; ΦNO: unregulated energy quantum yield of PSII; ΦNPQ: non photochemical quantum yield of PSII quenching; NPQ_t_: Non-photochemical quenching coefficient under steady-state illumination; qL: fraction of open PSII reaction centers; LEF: linear electron flow. ECStmAU: magnitude of electrochromic change; PSI_act_: photosystem I active centers; PSI_open_: photosystem I open centers; PSIox: photosystem I oxidized centers; PSIor: photosystem I reduced centers; gH + : proton conductance ATP synthetase; vH + : steady-state proton flux; F_v_/F_m_: photosystem II maximum quantum efficiency; F_m_: maximum chlorophyll fluorescence yield; F_o_: fluorescence yield in the dark; F_s_: steady-state fluorescence yield of chlorophyll; kP700: rate constant of electron transfer in P7; P700Da: oxidation probability of P700 after absorption of a photon; tP700: lifetime of p700 electron transfer in oxidized state; viP700: initial rate of steady-state electron transfer P700.

According to the cluster analysis carried out with the different physiological variables taken from the cacao clones, two typologies were found (Fig 7a). When all physiological variables were considered, the first two principal components explained 50% of the variability (Fig 7b). The first component explained 35.1% of the variability and separated clones ICS-95 and FLE-3 with higher values in NPQ_t_ and ΦNPQ traits, contrary to what was presented with clones such as FGH-4, LUKER-40, and IMC-67 that presented the highest values in F_m_, F_s_, F_v_/F_m_, PSI_AC_ and ΦNO. The second component explained 14.9% of the variability, highlighting the highest values in Chl_a_, Chl_T_, and Chl_b_ separating ICS-60; however, a lower value of Chl_a_ was associated with the highest values of PS1_ORC_, ΦII, LTD, and F_o_, highlighting clones FSA-13 and FEAR-5. According to the Monte-Carlo test, the clones explained 19% of the variance (P < 0.05). The contributing variables in components 1 and 2 are shown in Figs 6c and 6d, respectively. Table 1 shows each of the representative physiological characteristics of the cacao clones and their relationship to acclimatization.

**Table 1:** Physiological characteristics and acclimation traits of different cocoa clones under high temperature conditions in the Colombian Amazon.

Clone	Representative physiological characteristics	Acclimation
**FGH-4**	High SLA (specific leaf area). high F_v_/F_m_ (maximum quantum yield of PSII). high efficiency in electron transport rate (ETR). and high fraction of energy dedicated to qP (photochemical quenching).	Excellent ability to optimize light capture. maintain the structural integrity of PSII. and ensure efficient photosynthesis under heat stress.
**LUKER-40**	High SLA. low leaf temperature (LT). high efficiency in heat dissipation through ΦNPQ. and a good balance between light capture and energy dissipation.	Efficient leaf cooling strategy. reducing thermal stress and optimizing photosynthesis under high irradiance and elevated temperatures.
**LUKER-50**	High carotenoid content (antioxidant pigments). high heat dissipation (ΦNPQ). and protection of the photosynthetic apparatus against oxidative damage.	Effective protection against oxidative stress through antioxidant mechanisms and thermal dissipation. ensuring the stability of the photosynthetic apparatus.
**ICS-60**	Higher total chlorophyll content (Chl_t_). high efficiency in light capture. and ability to maintain high levels of ΦII (quantum efficiency of PSII).	High capacity for carbon fixation. maintaining photosynthetic activity and performance under thermal stress and high irradiance conditions.
**FSV-41**	Higher maximum quantum yield of PSII (F_v_/F_m_). high efficiency in electron transport rate (ETR). and a good balance between ΦII and ΦNPQ.	High photosynthetic efficiency even under thermal stress conditions. thanks to the ability to balance light capture and heat dissipation.
**IMC-67**	High efficiency in electron transport rate (ETR). good heat dissipation capacity through ΦNPQ. and stable F_v_/F_m_ values under thermal stress.	Resilience to thermal stress through energy compensation strategies. ensuring the functionality of the photosynthetic apparatus.
**ICS-95**	Increased NPQt and ΦNPQ (non-photochemical quenching). indicating a high capacity to dissipate excess light energy as heat.	Effective strategy to prevent oxidative damage under high irradiance conditions through thermal dissipation mechanisms that protect PSII.
**FLE-3**	High energy dissipation (NPQt and ΦNPQ). reduction in ΦII. and protective mechanisms of the photosynthetic apparatus through thermal dissipation.	Protection against oxidative damage through thermal dissipation. although with reduced capacity to maintain photosynthetic efficiency under adverse conditions.
**TSH-565**	Reduction in F_v_/F_m_ (<0.72). low ΦII values. and decreased overall photosynthetic efficiency under thermal stress.	Limited capacity to acclimatize to thermal stress, which affecting the functionality of the photosynthetic apparatus.
**EET-8**	Low F_v_/F_m_. ΦII. and ETR values. indicating severe damage to the photosynthetic apparatus and limited capacity to dissipate excess light energy.	Severe stress on the photosynthetic apparatus under adverse conditions. with limited ability to protect against oxidative damage and maintain photosynthesis.

**Fig 8:**
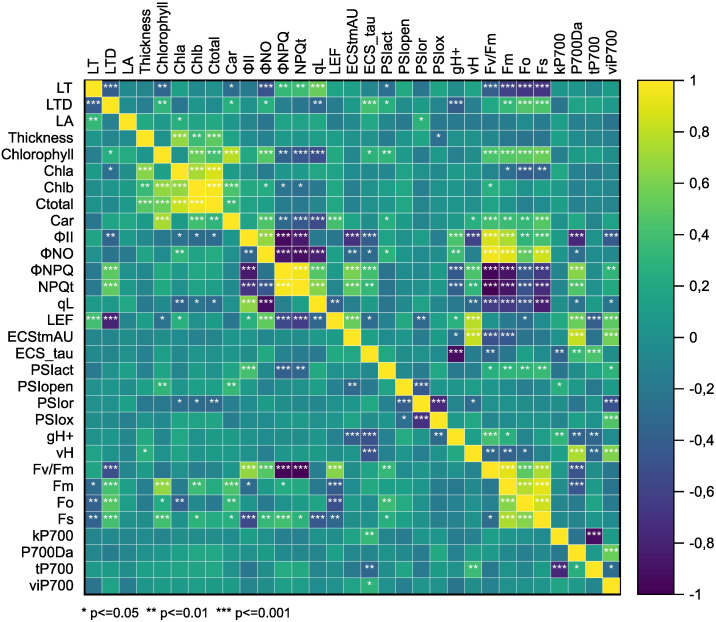
Pearson correlation coefficients between variables of energy use and distribution in the photosynthetic apparatus in different cocoa clones. Color gradient from green to red means positive and negative correlation between the different variables. *P<=0.05, **P<=0.01, ***P<=0.001. Diagonally down and diagonally up means the correlations at 06:00 h and 12:00 **h.** The acronyms of the variables are described in Fig 7.

When analyzing the incidence of temperature (LTD, Fig 8) on the functioning of the photosynthetic apparatus of cacao clones we found a negative effect on the amount of energy allocated to carbon fixation processes (ΦII), the fraction of open centers of PSII (qL) and the maximum quantum efficiency of photosystem II (F_v_/F_m_). However, this effect of the temperature increase affected the activation of energy dissipation traits in the form of heat (ΦNPQ, NPQ_t_) as the unregulated one (ΦNO). The increase in temperature had a positive effect on the linear flow of electrons (LEF), as well as on the PSI_open_ and PSI_or_. On the other hand, the fraction of energy used in photosynthetic apparatus processes presented a negative correlation with ΦNPQ, NPQ_t_, LEF, ECStmAU, ECS_tau, among others, and positive with F_v_/F_m_.

**Fig 9:**
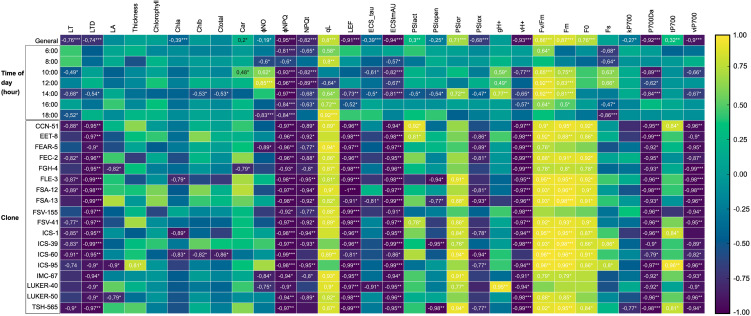
Heat map of Pearson correlation coefficients between photochemical quantum yield of photosystem II (ΦII) and different variables of energy use and distribution in the photosynthetic apparatus monitored at different times of the day (06:00, 08:00, 10:00, 12:00, 14:00, 16:00 and 18:00 hours) and evaluated in different cocoa clones. Colors represent direction and strength of correlation, *, ** and *** indicate significant correlations at 10, 5 and 1% probability level, respectively. The acronyms of the variables are described in Fig 7.

When relating the different variables that show the functioning of the photosynthetic apparatus with the fraction of energy dedicated to the photochemical quantum yield of PSII (ΦII), different positive and negative correlations were found in the different clones and time of day factors (Fig 9). For example, LA, leaf thickness, chlorophyll content, Chl_a_, Chl_t_, gH + , and F_s_ did not present any correlation with ΦII (P > 0.05). However, ΦII presented a high correlation (r > 0.8, P < 0.05) with F_v_/F_m_, F_m_, qL, F_o_, and PSI_or_. On the other hand, variables such as ΦNPQ, ECStmAU, vH, P700Da, LEF, viP700 presented a high negative correlation with ΦII (r > -0.9; P < 0.05; Fig 9). When analyzed in each of the hours sampled independently by the cacao clone, it was found that ΦNPQ was the variable that presented a negative correlation during all the times sampled, with the highest negative correlation between 12:00 h and 14:00 h (Fig 9). Likewise, variables such as NPQ_t_, ECStmAU, and P700Da presented a negative correlation from 10:00 h to 14:00 h with ΦII. However, F_v_/F_m_, F_m_, and gH+ positively correlated 10:00 h to 14:00 h with ΦII. On the other hand, some variables, such as ΦNO, qL, and F_s_, presented positive and negative correlations during the day with ΦII, and others did not correlate during the different times during the day with ΦII (Fig 9). At the cacao clone level, regardless of the time of day, it was found that variables such as LTD, ΦNPQ, LEF, ECStmAU, P700Da, and viP700 presented a negative correlation in all the clones evaluated, and NPQ_t_ and vH+ only presented no negative correlation for clones LUKER-40 and ICS-60, respectively, with ΦII. In the case of variables such as qL, PSI_or_, F_v_/F_m_, F_m_, F_o_ presented a positive correlation in most cacao clones with ΦII (Fig 9).

## 4 Discussion

To recall the study aim, we sought to compare the diurnal behavior of the photosynthetic apparatus—including Chl_a_ fluorescence, NPQ and PSI oxidation—across 18 cacao cultivars to identify physiological traits linked to photoprotection and recovery under high irradiance and thermal stress. Our results reveal cultivar-specific diurnal patterns: some clones exhibited sustained NPQ activation and increased PSI oxidation at midday with rapid recovery of photochemical activity in the afternoon, whereas others showed weaker protective responses and slower recovery. These contrasting responses suggest that coordinated NPQ and PSI oxidation dynamics under midday stress constitute useful functional markers for selecting cacao genotypes with greater tolerance to Amazonian diurnal extremes and provide mechanistic insight for breeding and agroforestry design.

### 4.1 Diurnal photosynthetic plasticity and photoprotection strategies among cacao clones under natural field conditions in the Colombian Amazon

In this present study for the first time the clones of cacao in the Colombian Amazon were investigated concerning the photosynthetic response under a natural typical day oscillation. The data clearly shows that energy dissipation pathways in the form of heat (ΦNPQ, NPQ_t_), the magnitude of the electrochromic shift (ECStmAU) and the probability of oxidation (P700Da) increases considerably at midday, while the fraction of open PSII reaction centers (qL) decreases at noon. This behavior in qL has been related to several studies where higher dissipation in heat pathways occurs when ΦII is reduced [43,44]. Similarly, it has been reported that P700, during the reduction or photooxidation cycle, absorbs excess energy and dissipates it as heat [45,46]. A decrease in photosynthetic efficiency induces oxidation of P700 in PSI [47]. Our study indicated that an increase in P700Da at midday is due to PSI oxidation, which causes a reduction in the photochemical rate. In this regard, it has been reported that P700 oxidation reduces intracellular carbon, decreases the photosynthetic rate, and increases photorespiration [47,48].

The clones IMC-67 and LUKER-40 stood out for presenting lower photoinhibition at noon, in parallel with lower NPQ activation. In contrast, clones ICS-95 and ICS-39 presented greater photoinhibition according to the F_v_/F_m_ data, possibly due to higher NPQ. This type of response is consistent with evidence of protective photoinhibition, caused by sustained NPQ [49]. This hypothesis is also corroborated by the rapid recovery presented by all materials after 16:00 h. Clones such as FGH-4, FLE-3, FSA-12, CCN-51, FSA-13, FSV-4, ICS-95, and LUKER-50 also stood out for a higher initial rate of steady-state P700 electron transfer (viP700), when reductions in the quantum yield of photosystem II were observed. This behavior highlights the plasticity of the cacao photosynthetic apparatus to survive under excess energy, despite its evolutionary origin. Similarly, a decoupling between PSII and PSI have been reported, where PSI maintains electron transfer as a compensatory strategy due to the low efficiency of PSII [47].

Increased chlorophyll content has been shown to enhance PSII efficiency under low light conditions [50]. In contrast, under high irradiance, plants activate photoprotective mechanisms such as increasing the xanthophyll content, a carotenoid pigment associated with non-photochemical quenching (NPQ) and antioxidative defense [18,51,52]. An increase in carotenoid content in response to varying light conditions, as observed in LUKER-50, was also reported by Suárez et al. [16], who found elevated carotenoid levels and a higher chlorophyll-to-nitrogen (Chl/N) ratio in cacao trees exposed to high radiation environments. According to Chen et al. [53], changes in the carotenoid-to-chlorophyll ratio may reflect adjustments in the proportion between light-harvesting antenna complexes and reaction centers, optimizing the use of available light resources according to environmental conditions. Additionally, certain carotenoids play a direct role in quenching reactive oxygen species, such as singlet oxygen, which is produced in large quantities within photosystem II under high light exposure. Thus, the dynamic responses in terms of carotenoid content in cacao leaves could improve not only light harvesting but also prevent membrane peroxidation by quenching ROS [54].

This study demonstrates that, over the course of a typical day, cacao plants activate temporal physiological adjustments related to stress defense and plasticity, with responses varying distinctly among the evaluated genotypes. Among the observed changes, clones EET-8, TSH-565, and FGH-4 exhibit higher initial fluorescence (F₀), while FGH-4—along with ICS-60 and ICS-1—also shows increased maximum fluorescence (Fₘ). Notably, clone FSV-41 presents the highest maximum quantum efficiency of photosystem II (F_ν_/Fₘ), indicating superior photochemical performance. According to Mathur and Jajoo [55], increases in these parameters correspond to plants activating the acclimation mechanisms of their photosynthetic machinery. In this context, the F_ν_/Fₘ parameter is widely recognized as a key indicator of stress in plants [56]. In healthy, unstressed plants, F_ν_/Fₘ typically averages around 0.80 [57], whereas values falling below 0.71 are indicative of chronic photoinhibition [58].

However, there is growing evidence that photodamage is not the main cause of photoinhibition and that the two processes are not equivalent. Several studies suggest that under natural conditions, continuous repair of the D1 protein and activation of non-photochemical quenching (NPQ) protective mechanisms help preserve the functionality of photosystem II (PSII). For example, reductions in F_v_/F_m_ are attributed to downregulation of PSII in the NPQ pathway rather than irreversible photodamage [49]. In our study, incomplete relaxation suggests sustained NPQ in darkness. An inverse relationship between NPQ_t_ and F_v_/F_m_ was observed. In addition, all clones except IMC-67, TSH-565, and EET-8 had F_v_/F_m_ greater than 0.72, indicating high photosynthetic performance under typical daytime conditions in the Amazon [49,59]. The diurnal study revealed that variables such as LT, LTD, NPQ_t_, and ΦNPQ increased until midday, while F_v_/F_m_, qL, and ΦII decreased. in the morning hours (06:00 h to 10:00 h), cocoa plants are more efficient from a photosynthetic point of view, capturing more light in the antennal complexes and absorbing CO_2_ without reaching a saturation point. It is worth mentioning that radiation levels reached around 400 μmol m^-2^ s^-1^, a level suitable for optimal physiological behavior [60–63]. At midday, higher leaf temperature, lower ΦII, and maximum dissipation (NPQ_t_, ΦNPQ) were observed, with stable ΦNO. The ICS-95 and IMC-67 clones showed the greatest reduction in F_v_/F_m_ and ΦII, while peaks in NPQ were consistent with reversible protective photoinhibition [60–64]. Thus, the photoinhibition presented by cacao at noon in the current study should most likely be reversible, determined by the reduction of F_m_ generated by the sustained NPQ state. In general, clones with a lower decrease in F_v_/F_m_ and moderate NPQ at midday, together with rapid recovery, show greater resilience to peaks in irradiance and heat.

According to different studies, high temperatures are one of the limiting factors in cacao [31], affecting plant physiology, including initial increase in stomatal aperture followed by a water loss [59–63], limiting CO_2_ assimilation, decreased chlorophyll synthesis, alterations in electron transport rate and loss of enzymatic activity [64–66]. The decreases in photochemical variables and fluctuations in chlorophyll content in the present study could be partially related to the high temperatures that ranged between 28–35°C in the evaluated conditions. However, it is important to note that the temperature differences in the present study were accompanied by changes in light intensity and vapor pressure deficit within the framework of a typical day and therefore the interactive potential of other abiotic processes in the responses presented by the cocoa plants cannot be ruled out. Moreover, increased stomatal conductance at high temperatures has been reported to favor increased photosynthetic rates in short heat waves when water is abundant in the soil; prolonged episodes accompanied by water deficit cause a disadvantage [59]. Also, under adverse combined conditions, damage to proteins and enzymes in chloroplasts and mitochondria may occur, which could lead to cell death under prolonged exposure [67].

In the literature it is reported that the increase in temperature also had a negative effect on photochemical processes, reducing the ΦII, qL, PSI_ox_, F_v_/F_m_, F_m_, F_0_ and induce energy dissipation in the form of heat. This behavior has been exposed by Mathur et al. [68], Mathur and Jajoo [55], and Mathur et al. [56] in their studies, where they state that high temperatures cause closure in the PSII reaction centers and most of the absorbed energy is dissipated as heat. In the present study several physiological traits varied at noon, when the temperature was higher, such as: increment of linear electron flow, reduction of photosystem I reaction centers, changes in proton conductance of ATP synthase, steady-state proton flux, probability of P700 oxidation after photon absorption, and increase in the initial rate of steady-state electron transfer. In this regard, it has been reported that the combined effects of light and temperature in tropical regions can cause photoinhibition processes of PSI and PSII [69,70]. In addition, the increase of electron flux from PSII to PSI [71] combined with reduced proton conductance in the thylakoid membrane [72] could be associated with photoinhibition in PSI [73].

To better understand the physiological responses under complex environmental conditions, such as those associated with field experiments, it is necessary to resort to integrative analysis approaches. Multivariate analysis indicate interesting relationships specific to the various cacao clones, among them, photosynthetic pigments were associated with lower leaf temperature (LTD), greater presence of photosystem I reaction centers in active, open state, higher values of minimum fluorescence, maximum fluorescence and consequently higher quantum efficiency of photosystem II, which translates into higher quantum yield of photosystem II (ΦII) and lower energy dissipation in the form of heat. Xiong et al. [74] reported that chlorophyll concentrations are influenced by time of day as a function of radiation used. In fact, previous reports indicate that F_v_/F_m_ dynamics during the day are associated with negative changes in chlorophyll content [75].

While our study provides valuable information on the diurnal photosynthetic responses of cacao clones under natural field conditions, we acknowledge that several potential confounding factors may have influenced our results and merit careful consideration. The experimental design, although robust in capturing temporal variability through six daily measurement points over three consecutive days, did not completely control for the variability or effect that soil moisture might cause. Previous research in Amazonian cacao agroforestry systems has demonstrated that soil properties can explain up to 33% of the total variance in productivity, with soil texture, structural stability, and water retention capacity significantly affecting plant performance [76]. Variations in soil moisture content across experimental plots could have contributed to differential stress responses among clones.

### 4.2 Functional traits for Cacao clone selection: Integrating SLA and chlorophyll fluorescence for breeding and agroforestry design in the Colombian Amazon

In previous reports, a key trait underlying cacao productivity is variation is specific leaf area (SLA), which showed high variability across clones—an important consideration for clone selection [77]. Prior studies have linked greater SLA to higher productivity, independent of environmental conditions, suggesting this trait may confer a general adaptive advantage [78]. Indeed, SLA can be influenced by multiple factors, including system management. For instance, agroforestry practices have been shown to increase SLA by 9.5% to 24.4% under shade compared to full sun conditions [29,79]. While higher SLA might initially be assumed to aid photoprotection under high light, shade-adapted species like cacao often develop increased SLA to optimize light capture in low irradiance environments [80]. Interestingly, high SLA can also serve to reduce water loss via lower transpiration rates [78], yet this seems contradictory, as plants under intense light typically face greater water stress, where lower SLA would be expected. These contradictions highlight the complexity of SLA as a functional trait and emphasize the need for deeper investigation into its role in morpho-physiological responses to environmental variability.

In this study, the genotypes LUKER-40 and FGH-4 exhibited the highest SLA values, suggesting greater leaf expansion relative to biomass investment. Although SLA is generally responsive to light conditions [81], previous work comparing clones such as ICS-95 and LUKER-50 across light regimes found no significant differences in this trait, indicating that genetic factors may override environmental influences in some circumstances [17]. Notably, clones with higher SLA did not necessarily develop thicker leaves, indicating that SLA alone does not determine anatomical structure and consequently the total photosynthetic surface area available for light interception. For instance, ICS-60 exhibit the thinnest leaves along with the lowest concentrations of chlorophyll *a*, *b*, and total chlorophyll (Chl_t_), implying reduced potential for light absorption [32]. This inference is supported by chlorophyll a/b ratio data, which further suggests suboptimal chloroplast structure and function in this clone [16,82]. Altogether, these findings reinforce the importance of integrating SLA with other physiological and biochemical markers when assessing cacao clones for breeding or agroforestry designing purposes.

In this context, chlorophyll fluorescence techniques allowed the phenotyping of cacao clones, identifying those with better performance under typical day conditions in the Colombian Amazon. In this study, we highlighted clones such as LUKER-40, FGH-4, FEAR-5, FSV-155, IMC-67, and LUKER-50, which exhibited physiological mechanisms associated with more efficient use of light energy for photochemical pathways and a reduction in energy dissipation as heat, suggesting less susceptibility to photoinhibition. This behavior is associated with a higher presence of chlorophyll, and open centers of photosystem I, which underscores their high capacity for light capture and protection against photoxidative damage, thus making them promising candidates for implementation strategies in agroforestry systems in the Colombian Amazon. In contrast, ICS-95, ICS-39, FSA-13, and FLE-3 clones increase heat dissipation pathways and reduce photochemical pathways. However, further clarification of these behaviors across different developmental stages is required.

## 5 Conclusion

Our study demonstrated how diurnal temperature changes affect the photosynthetic capacity of cocoa in its juvenile stage. Cocoa clones in adverse conditions at midday activate various physiological responses to protect their photosynthetic apparatus and maintain their metabolic activity, key aspects that can be exploited in genetic improvement programs. Among these responses, heat dissipation through non-photochemical mechanisms (ΦNPQ and NPQ_t_) stands out, allowing excess light energy to be released in the form of heat, thus preventing damage to the photosynthetic apparatus (PSII). Clones such as LUKER-50, ICS-95, ICS-39, and FLE-3 stood out for their high capacity for non-photochemical dissipation (NPQ) and for maintaining protective photoinhibition at midday. In addition, LUKER-50 stood out for its high carotenoid content, which can act as antioxidants and protect the photosynthetic apparatus from oxidative stress. The LUKER-40 clone stood out for its reduction in leaf temperature (LT), optimizing transpiration and heat dissipation, which makes it more efficient at high irradiance. Similarly, maintaining photosynthetic efficiency (F_v_/F_m_ and ETR) is crucial for PSII to maintain electron transport and quantum efficiency under thermal stress. Clones such as FGH-4, FSV-41, and IMC-67 stand out for maintaining high F_v_/F_m_ and ETR values even at elevated temperatures. Finally, the ICS-60 clone stands out for its higher total chlorophyll content (Chl_t_), allowing for greater light capture and stability in photosynthetic performance even under heat stress conditions. The selected clones represent a desirable genetic base for expanding cocoa genetic resources in the Amazon, due to their good capacity to tolerate and efficiently use light energy. Similarly, physiological responses and key strategies in the acclimatization of cocoa clones in the region are evident. Our results also suggest the need to expand the analysis of cocoa clone adaptation under field conditions to find materials that demonstrate greater photosynthetic acclimatization in contrasting environments.

## Supporting information

S1 Fig Images of chlorophyll fluorescence parameters taken in fully developed leaves at predawn. (JPG)

S2 Fig Images of the Y(II) parameter obtained by IMAGING-PAM; the change in the color gradient from violet to red indicates the change in value from higher to lower for each variable. (JPG)

S3 Fig Images obtained with IMAGING-PAM; (a) qP; (b) Y(NPQ); (c) qN; the change in the color gradient from violet to red indicates the change in value from higher to lower for each variable. (JPG)

S4 File Physiological variables. (XLSX)
