# Supplementary figures and images for: Understanding mechanistic responses underlying diurnal photoprotection and photosynthetic plasticity among cacao genotypes under natural amazonian field conditions
Source: PLoS One. 2026 Jun 11;21(6):e0351655. doi: 10.1371/journal.pone.0351655 (PMC13257972; doi:10.1371/journal.pone.0351655)

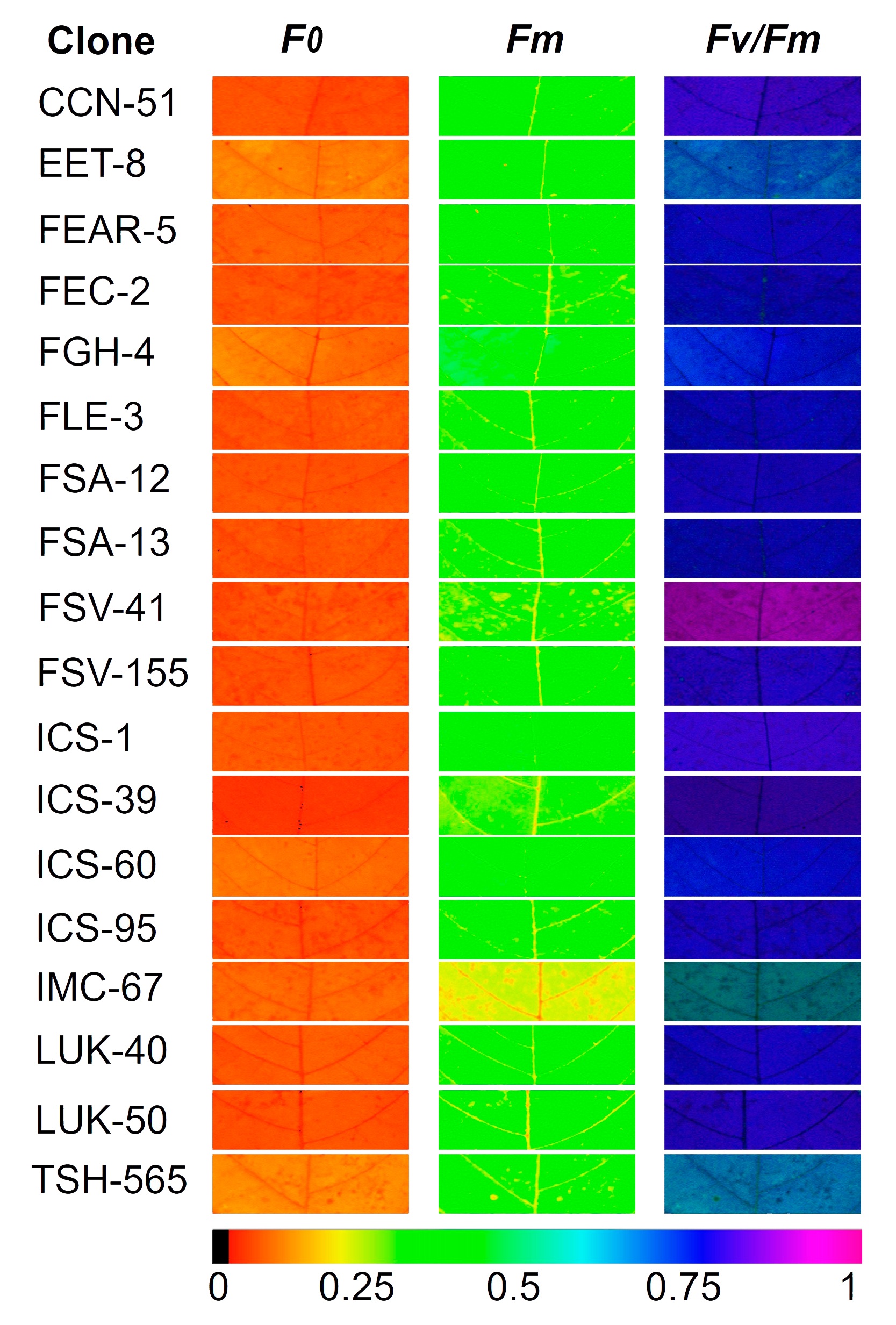

Supplement: S1 Fig — (JPG) [file pone.0351655.s001.jpg]

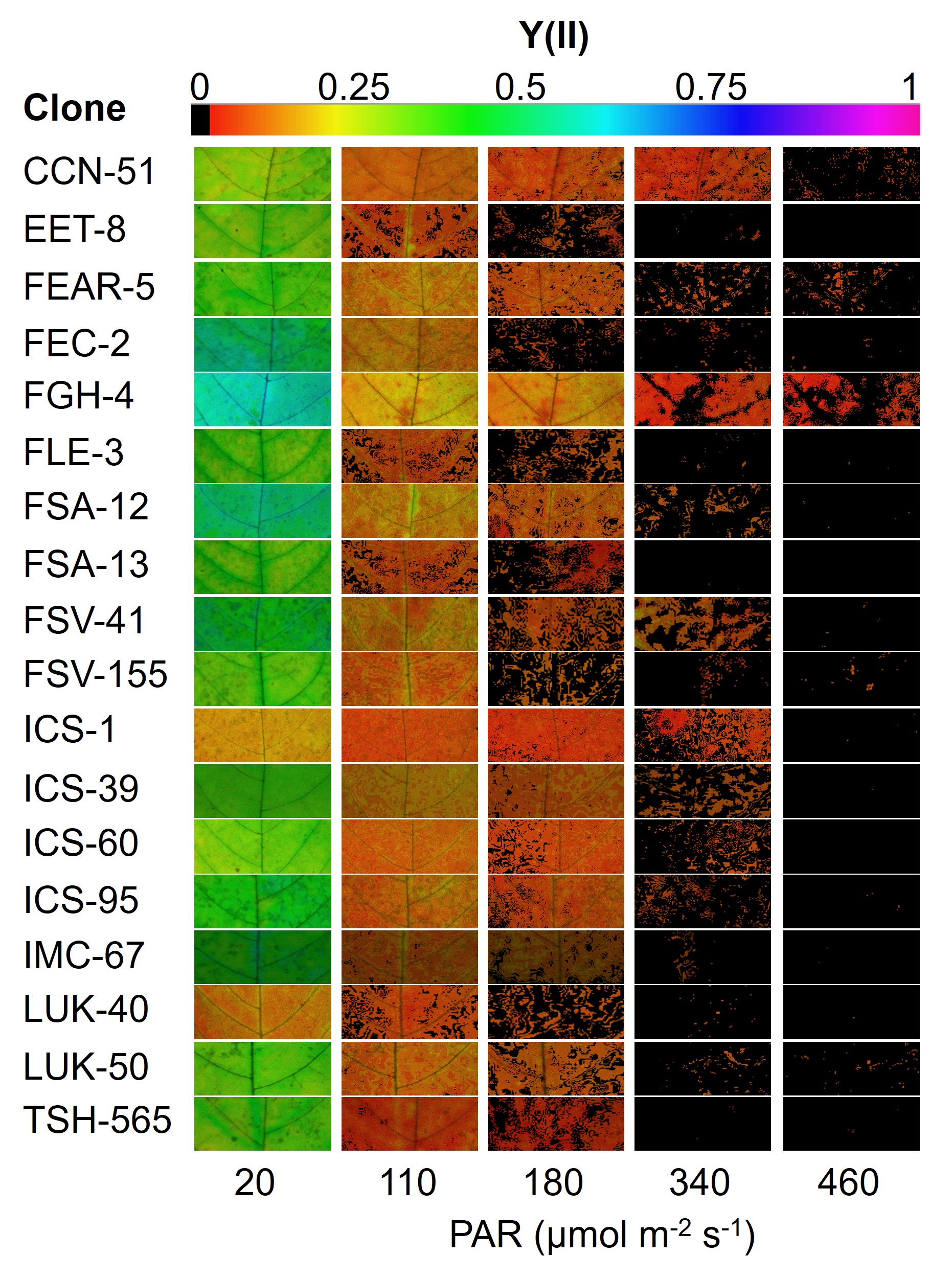

Supplement: S2 Fig — (JPG) [file pone.0351655.s002.jpg]

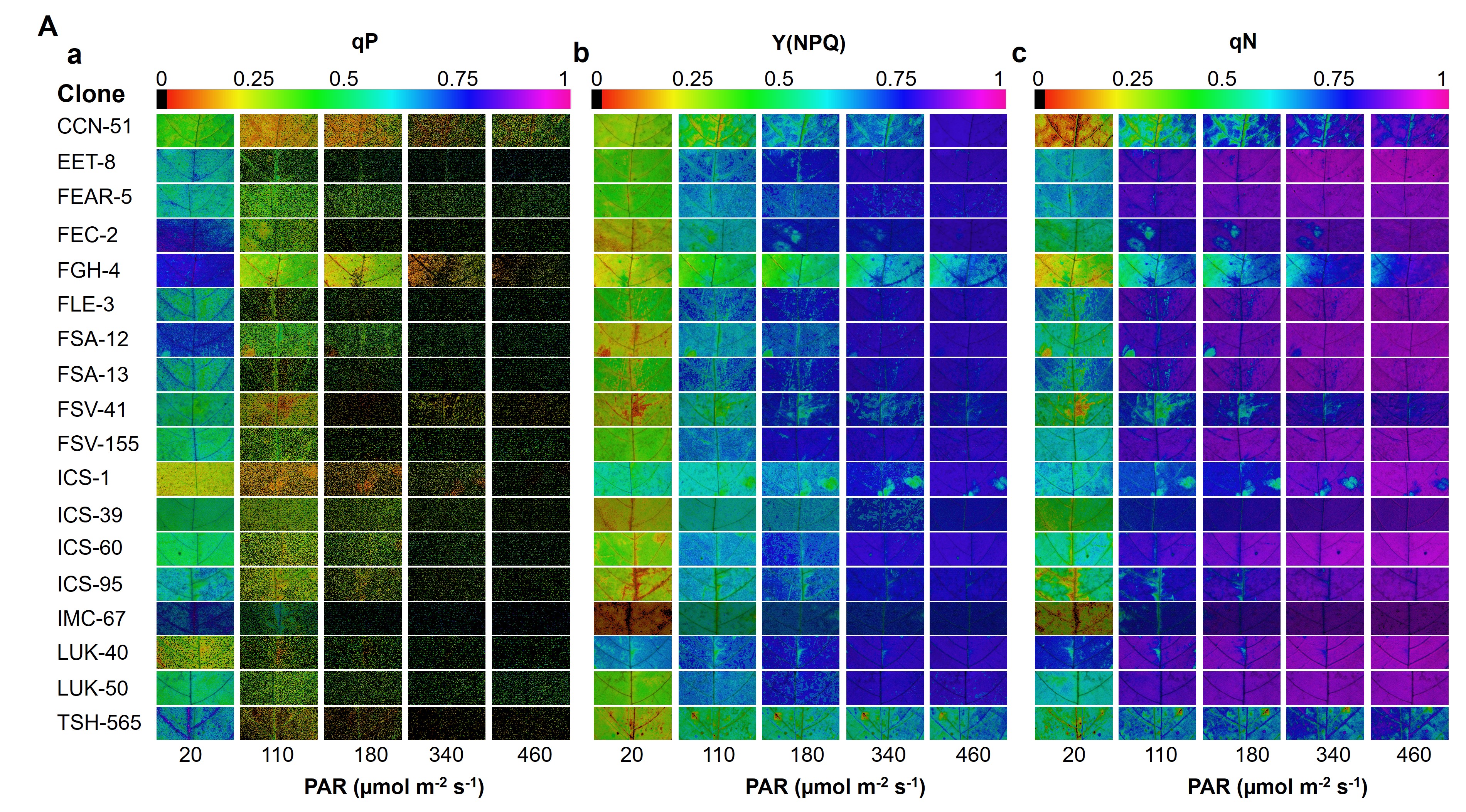

Supplement: S3 Fig — (JPG) [file pone.0351655.s003.jpg]
